# Conferring NiTi alloy with controllable antibacterial activity and enhanced corrosion resistance by exploiting Ag@PDA films as a platform through a one-pot construction route

**DOI:** 10.1016/j.heliyon.2024.e34154

**Published:** 2024-07-09

**Authors:** Ying Li, Yongkui Yin, Luxin Li

**Affiliations:** a College of Life Science, Mudanjiang Medical University, Mudanjiang, 157011, Heilongjiang Province, PR China; b School of Health Management, Mudanjiang Medical University, Mudanjiang, 157011, Heilongjiang Province, PR China

**Keywords:** Nickel-titanium alloy, Silver nanoparticles, Polydopamine, Corrosion resistance, Antibacterial platform

## Abstract

The lack of antibacterial activity and the leaching of Ni ions seriously limit the potential applications of the near equiatomic nickel-titanium (NiTi) alloy in the biomedical field. In this study, a silver nanoparticles (Ag NPs) wrapped in a polydopamine (Ag@PDA) film modified NiTi alloy with controllable antibacterial activity and enhanced corrosion resistance was achieved using a one-pot approach in a mixed solution of AgNO_3_ and dopamine. The controllable antibacterial activity could be achieved by adjusting the initial concentration of dopamine (C_dop_), which obtained Ag@PDA films with varying thickness of polydopamine layers coated on Ag NPs, thereby conferring different levels of antibacterial activity to the modified NiTi alloy. *In vitro* antibacterial ratios (24 h) of Ag@PDA film-modified NiTi alloy against *E.coli* and *S.aureus* ranged from 46 % to 100 % and from 42 % to 100 %, respectively. The release curves of Ag ions indicated the persistent antibacterial effect of Ag@PDA film-modified NiTi alloy for at least 21 days. Moreover, *in vitro* cytotoxicity and in *vivo* implantation tests demonstrated the satisfactory biosafety of the Ag@PDA film-modified NiTi alloy when used as bioimplants. This research offers valuable insight into meeting various antibacterial demands for NiTi alloy implantations and highlights the potential of Ag-containing film-modified biomaterials in addressing different types of infections induced by implantations.

## Graphical abstract

**Figure:**
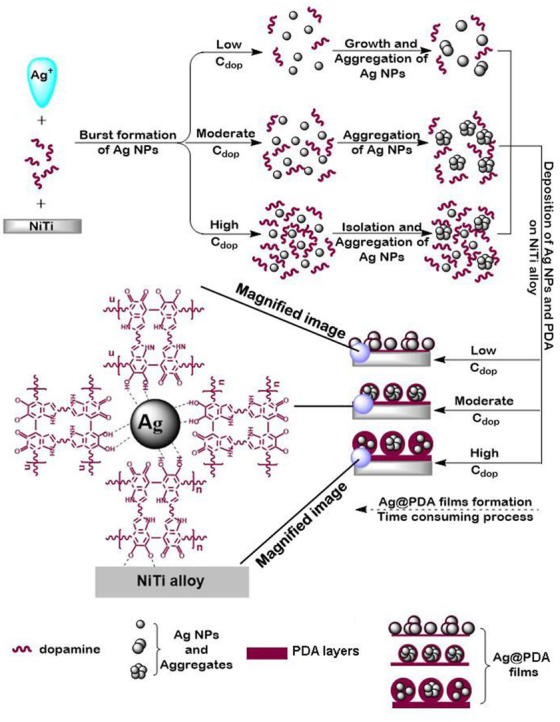

## 1 Introduction

Due to traffic accidents, diseases, and natural disasters, the clinical demand for artificial implants and surgical devices has been increasing in recent years [1,2]. Among the various metal bioimplants, the near equiatomic nickel-titanium (NiTi) alloy has gained wide bioapplications due to its specific shape memory effect and superelasticity [3,4]. However, its poor antibacterial properties may lead to postoperative infections, potentially resulting in implant failure, delayed healing, and other post-surgical complications [5,6]. Additionally, the high Ni content in the alloy raises considerable concerns, as excessive leaching of Ni ions could pose a high risk of causing allergic reactions and promoting carcinogenesis [[7], [8], [9]]. The need for antibacterial ability to address infections resulting from various implantations is specific [10]. Therefore, the development of a controllable antibacterial platform on NiTi alloy with enhanced corrosion resistance is crucial to prevent postoperative infections and Ni ions leaching.

The traditional approach to preventing or treating infections caused by implantations involves systemic antibiotic use or modifying antibiotics onto therapeutic carriers. However, the excessive use of antibiotics leads to the development of antibiotic-resistant bacteria, posing a serious threat to public health [11,12]. Consequently, metal antibacterial agents such as Ag, Cu, and Zn have garnered significant attention due to their low incidence of antibiotic resistance [[13], [14], [15]]. Among these, Ag nanoparticles (NPs) exhibit high antibacterial efficiency [16,17] against a broad spectrum of bacteria and have found applications in catheter coatings [18], wound dressings [19], antibacterial band gels [20], and more. Therefore, incorporating Ag NPs for surface modification of biomaterials represents an ideal strategy preventing implant infections. Numerous efforts have been directed towards fabricating Ag NPs modified biomaterials, employing techniques such as electrochemical deposition [21], physical vapor deposition [22], pulsed filtered cathodic vacuum arc deposition [23], and sputtering deposition [24]. However, the Ag NPs modified layers constructed on bioimplants often fail to achieve controllable antibacterial activities that meet diverse demands of various implantations. Moreover, the further application of these techniques is hindered by their complex synthesis procedures, intricate preparation equipment, and high cost.

The construction of polydopamine (PDA) films has been demonstrated as a direct technology for modifying material surface by immersing them into a mild alkaline dopamine solution [[25], [26], [27]]. Furthermore, immersing PDA film-modified material into an AgNO_3_ solution, allows the ample catechol groups on the PDA films to reduce Ag ions to Ag NPs [28,29], resulting in the formation of Ag NPs loaded PDA (Ag/PDA) films. The loading content of Ag NPs can be regulated by adjusting the initial concentration of AgNO_3_ (C_AgNO3_) or the immersion time [[30], [31], [32]], enabling the Ag/PDA films to possess controllable antibacterial activities. Additionally, PDA films can improve the corrosion resistance of the modified materials [[33], [34], [35]]. Despite Ag/PDA films loaded with sufficient Ag NPs being capable of exhibiting controllable antibacterial activities and enhanced corrosion resistance, the time-consuming preparation of PDA films and the multi-step preparation procedures of the Ag/PDA films still limit their further applications. Therefore, a simpler route is necessary to combine the advantages of Ag NPs and PDA films to achieve controllable antibacterial activities and enhanced corrosion resistance.

In previous research [36], an Ag NPs wrapped in a PDA (Ag@PDA) film modified NiTi alloy with excellent antibacterial activity and enhanced corrosion resistance was achieved through a simple one-pot route in a mixed solution of dopamine and AgNO_3_. In this study, we aim to construct Ag@PDA films capable of conferring NiTi alloy with controllable antibacterial activities and enhanced corrosion resistance by adjusting the initial C_dop_ in the mixed solution. The *in vitro* antibacterial ratios (24 h) against *Escherichia coli* (*E.coli*) and Staphylococcus aureus (*S. aureus*) range from 46 % to 100 % and from 42 % to 100 %, respectively. Furthermore, assessments of *in vitro* cytotoxicity and *in vivo* implantation demonstrate the good biosafety of Ag@PDA film-modified NiTi alloy, indicating its potential application in various implantations.

## 2 Materials and methods

### 2.1 Preparation methods

The equiatomic NiTi plates, measuring 10 mm × 10 mm × 2 mm, were ground and mirror-polished using 1.5 μm diamond paste. Subsequently, the polished NiTi plates underwent successive ultrasonic washing with acetone, ethanol, and deionized water for 10 min before preparing the PDA-based films. The coating solution, comprising 20 mL, was prepared in a 100 ml beaker and consisted of a mixture of 40 mM AgNO_3_ (Sigma-Aldrich) and predetermined concentration of dopamine hydrochloride (Sigma-Aldrich). The pH value was adjusted to 8.5 using Tris base (Sigma-Aldrich). The cleaned NiTi plates were immersed directly in the above solution and then placed in a photophobic water bath constant temperature oscillator (SHA-BA, Shanghai Guning Instrument Co., Ltd) with gently vibration for 12 h at 25 °C. The cyclotron amplitude was set at 20 mm and the rotation rate was controlled at 50 rpm/min. Finally, the samples were thoroughly cleaned in deionized water with ultrasonication for 5 min and dried in a vacuum at 40 °C. The predetermined C_dop_ was set at 1, 2, 3, 4, and 5 mg/mL, and the corresponding films on NiTi alloys were termed Ag@PDA*m*, where “*m*” represents the value of C_dop_.

### 2.2 Characterizations of the films

The surface morphology and microstructure of polished NiTi alloy, PDA film-modified NiTi alloy, and Ag@PDA film-modified NiTi alloy were investigated using field emission scanning electron microscopy (FESEM, S4800, Hitachi) and transmission electron microscopy (TEM, JEOL-2100, JAPAN), respectively. The chemical composition and function groups of the films were determined by X-ray photoelectron spectroscopy (XPS, Bruke, USA) and Fourier transform infrared spectroscopy (Micro-FTIR, Thermo Fisher), respectively. Films of Ag@PDA*1*, Ag@PDA*2* and Ag@PDA*5* were scraped off for TEM observation and FTIR testing. The surface roughness and topological structure of Ag@PDA film-modified NiTi alloy were characterized using Atomic Force Microscopy (AFM, Bruker, USA) in tapping mode.

### 2.3 The corrosion tests and ions release behavior

The corrosion resistance of polished NiTi alloy, PDA film-modified NiTi alloy, and Ag@PDA film-modified NiTi alloy was tested in Hank's solution (containing 8.00 g/L NaCl, 0.4 g/L KCl, 0.06 g/L MgSO_4_·7H_2_O, 0.06 g/L NaH_2_PO_4_·2H_2_O, 0.35 g/L NaHCO_3_, 1.0 g/L Glucose, 0.6 g/L KH_2_PO_4_, 0.1 g/L MgCl_2_·6H_2_O, 0.14 g/L CaCl_2_, and PH = 7.4) at 37 °C. The work station used was the CHI 660A electrochemical system (CH Instruments, USA), with a saturated calomel electrode (SCE) as the reference electrode and a Pt foil as the auxiliary electrode. Samples with a surface exposed area of 1 cm^2^ were used as the working electrode and exposed to Hank's solution for 7200 s to obtain a steady open circuit potential (OCP). Subsequently, the polarization scan began at a potential below OCP and continued to 600 mV towards the anodic direction at a scanning rate of 0.5 mV/s.

The release behavior of Ag ions and Ni ions was evaluated by incubating each sample in 5 ml of PBS solution in darkness at 37 °C. The PBS solutions were refreshed and collected at 1, 4, 7, 14, 21, and 28 days. The concentration of the released Ag and Ni ions was determined using inductively coupled plasma optical emission spectrometry (ICP-OES, Leeman, USA). Each experimental group consisted of three samples.

### 2.4 *In vitro* antibacterial assessment

The *in vitro* antibacterial activities of polished NiTi alloy, PDA film modified NiTi alloy, and Ag@PDA film-modified NiTi alloy (n = 3) were evaluated using the bacterial counting method as per the National Standard of China GB/T 2591 and with some modifications. *Escherichia coli* (*E. coli*, a Gram-negative organism) and *Staphylococcus aureus* (*S. aureus*, a Gram-positive organism) suspended separately in Luria-Bertani (LB) medium at a concentration of 10^6^ CFU mL^−1^ were utilized as experimental strains. Typically, 50 μl of bacterial suspension was dispensed onto the surface of each sterilized sample in a 24-well plate. The samples was then covered with a sterilized polyethylene film (1 cm × 1 cm) and incubated for 24 h at 37 °C under humid conditions. Subsequently, each sample along with its bacterial suspension was transferred into a sterilized centrifuge tube containing 5 mL of PBS and vortexed for 5 min to detach the bacteria from the sample surface. Following a series of dilutions, 100 μl of each diluted bacterial solution was evenly spread on solidified nutrient agar plates and incubated at 37 °C for 18 h. The visible colonies were then counted, and the antibacterial ratio (*AR*) was calculated using the formula:

$$AR=\frac{{CFU}_{control}-{CFU}_{sample}}{{CFU}_{control}}\times 100\%$$

where *CFU*_*control*_ is the average number of bacteria of control group, and *CFU*_*sample*_ represents the average number of bacteria treated by the sample. Each group included three parallel samples.

### 2.5 *In vitro* cytotoxicity test

The cytotoxicity of polished NiTi alloy, PDA film-modified NiTi alloy, and Ag@PDA film-modified NiTi alloy was evaluated using Cell Counting Kit-8 (CCK8). Initially, Human bone mesenchymal stem cells (*hBMSCs*) sourced from the Stem Cell Bank of Chinese Academy of science were cultured in DMEM (Gibco), supplemented with 10 % fetal bovine serum and 1 % antimicrobial penicillin and streptomycin within an incubator set at 37 °C with 5 % CO_2_. Subsequently, 1 mL of the cell suspension was seeded onto each sample in a 24-well plate with a density of 5 × 10^4^ cells/well. Following co-cultivation for 1, 3, and 5 days, 50 μL of CCK8 solution was added to each well, and the plate was further incubated for 4 h. Afterwards, 100 μL of the resulting medium from each well was transferred to a 96-well plate. DMEM served as the negative control, while DMEM with 10 % dimethylsulfoxide (DMSO) acted as the positive control. The optical density (OD) of the solution was then measured at 570 nm using a microplate reader (Molecular Devices, M3, USA), with a reference wavelength of 630 nm. Triplicate parallel tests were conducted for each condition.

### 2.6 Subcutaneous implantation

Male Institute of Cancer Research (ICR) mice (weight: 25–30 g; age: 6–8 weeks) were procured from Beijing Vital River Laboratory Animal Technology Co., Ltd. (Beijing, China). All animal experiments adhered to the guidelines set forth by the Ethics Committee on Animals of Mudanjiang Medical University (Mudanjiang, China) (approval number: IACUC-20220516-9). Twenty mice (10–12 weeks old) were randomly assigned to four groups: NiTi, PDA (represents PDA film modified NiTi alloy), Ag@PDA (based on the findings of our previous research, Ag@PDA film modified NiTi alloy constructed under fixed conditions of C_dop_ at 2 mg/mL, C_AgNO3_ at 40 mM, and a coating time of 12 h, exhibited excellent antibacterial activity and enhanced corrosion resistance, therefore, Ag@PDA*2* was selected for Subcutaneous implantation), and control (no samples were implanted). In each group, two longitudinal incisions of 1 cm were made on the bilateral backs of each mouse. Subsequently, the corresponding samples (5 cm × 5 cm × 5 cm) were implanted, and the skin was then closed.

### 2.7 Biological security evaluation

After 30 days of treatment, the mice were euthanized, and the major organs (heart, liver, spleen, lung, and kidney) were removed from the corpses and then fixed in 4 % paraformaldehyde. Subsequently, H&E staining was conducted to determine if there were any significant pathological changes or organ damage.

## 3 Results and discussion

### 3.1 Composition and surface morphology

**Fig. 1:**
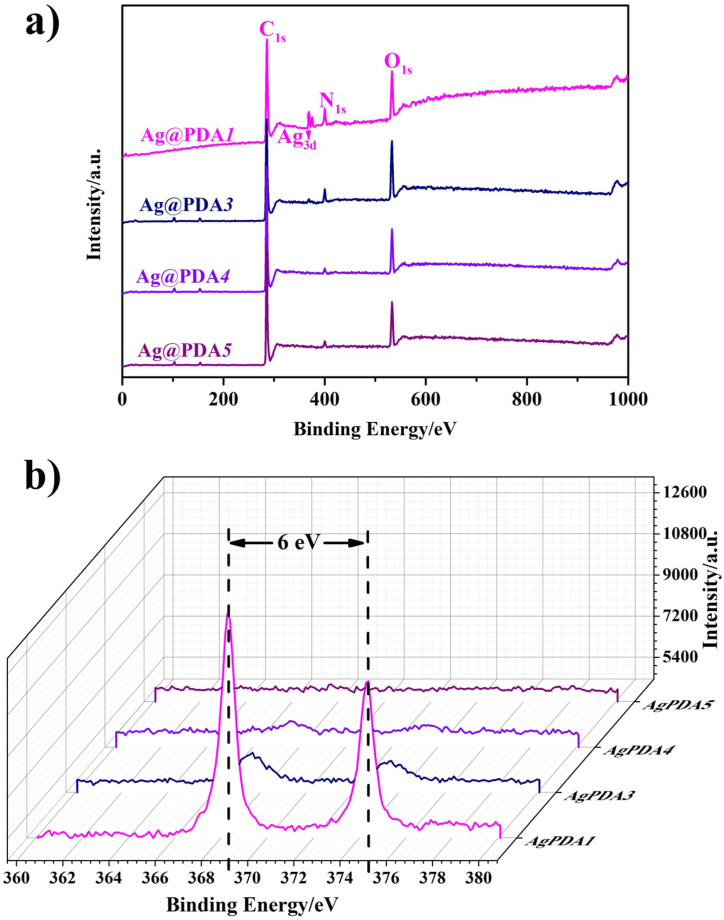
XPS spectra of the Ag@PDA film-modified NiTi alloy constructed by adjusting C_dop_, (a) Full spectra and (b) high-resolution spectra of Ag@PDA*1*.

Fig. 1 depicts the XPS spectra of the Ag@PDA film-modified NiTi alloy, constructed by adjusting C_dop_. In our previous report, the coating of PDA film on NiTi alloy, led to the emergence of the N_1s_ peak, while the typical characteristic peaks of NiTi alloy, such as 456 eV of Ti_2p_, 564 eV of Ti_2s_, and 850 eV of Ni_2p_, disappeared [36]. Compared to PDA film-modified NiTi alloy, Ag@PDA*4* and Ag@PDA*5* exhibit similar characteristic peaks, such as C_1s_, N_1s_, and O_1s_ (Fig. 1a). The other Ag@PDA film-modified NiTi alloy display the characteristic peaks of PDA film and Ag_3d_ (368.15 eV of Ag_3d5/2_ and 374.15 eV of Ag_3d3/2_), indicating the coexistence of metallic Ag and PDA (Fig. 1a and b). From Table S1, the corresponding Ag content ranging between 0.89 at% to 0.3 at% can be obtained by adjusting the C_dop_ from 1 mg/mL to 3 mg/mL. However, the Ag contents of Ag@PDA*4* and Ag@PDA*5* were less than 0.1 at%, which may be attributed to the rare Ag in the penetration depth of XPS [37].

**Fig. 2:**
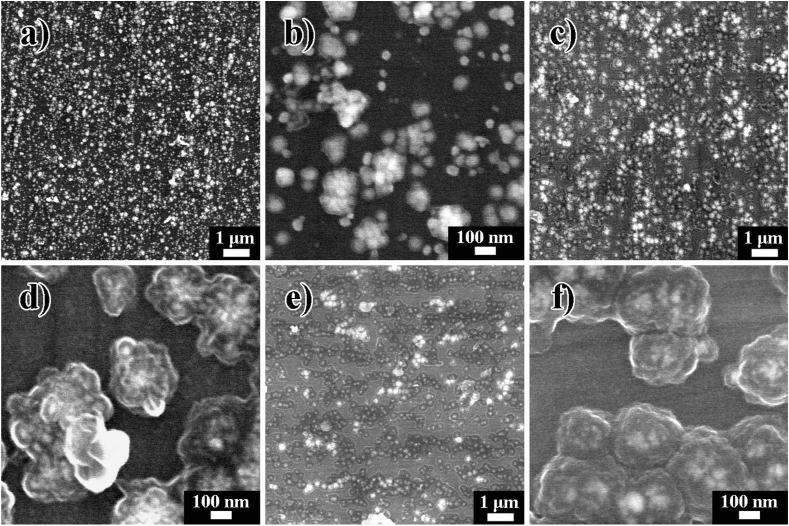
Surface morphology of the Ag@PDA film-modified NiTi alloy constructed by adjusting C_dop_, a) and b) Ag@PDA*1*, c) and d) Ag@PDA*3*, e) and f) Ag@PDA*4*, respectively.

The surface morphological evolution of the Ag@PDA film-modified NiTi alloy constructed by adjusting C_dop_ is depicted in Fig. 2. In our previous report, the polished NiTi possessed a relatively smooth surface (1.67 nm). In comparison, the PDA film-modified NiTi alloy exhibited a surface roughness of 4.74 nm, displaying some humps on its surface. Upon adding AgNO_3_ (40 mM) into a 2 mg/mL of dopamine solution, spherical aggregates with an average diameter of 245 nm were distributed on the surface of Ag@PDA*2*. These aggregates were consisted of NPs adhered to each other by some substance [36]. As shown in Fig. 2a and b, when adjusting the C_dop_ to 1 mg/mL, isolated or aggregated NPs with an average diameter of 73 nm can be observed on Ag@PDA*1* (Fig. S1a), but the adhered substance among the NPs is less than that of Ag@PDA*2*. Upon further increasing the C_dop_ to 3 mg/mL, aggregates with an average diameter of 267 nm are presented on Ag@PDA*3* (Fig. 2c and d, and Fig. S1b). The adhered substance among the NPs of the aggregates is further increased compared to Ag@PDA*2*. When the C_dop_ is increased to 4 mg/mL and even to 5 mg/mL, spherical chunks present on Ag@PDA*4* (Fig. 2e and f) and Ag@PDA*5* [36], respectively, with average diameters of 290 nm and 390 nm(Fig. S1c and Fig. S1d). Meanwhile, the surface roughness of Ag@PDA film-modified NiTi alloy increased with increasing the C_dop_, such as 19.6 nm for Ag@PDA*1*, 30.9 nm for Ag@PDA*3*, 40.3 nm for Ag@PDA*4*, and 74.9 nm for Ag@PDA*5* (Fig. S2).

Fig. S3 shows the surface chemical constituents of NiTi alloy, PDA, and Ag@PDA film-modified NiTi alloy obtained by EDAX. As shown in Fig. S3a, the main surface constituents of NiTi alloy are Ti, Ni, O, with minor amount of N and C likely due to unavoidable air pollution. In contrast, a significant increase in N and C is observed in the spectrum of PDA (Fig. S3b), which primarily attributed to the formation of the PDA film on the modified NiTi alloy. When AgNO_3_ was introduced into the dopamine solution, Ag peaks appeared in the EDAX spectrum of Ag@PDA film-modified NiTi alloy. Futhermore, as the C_dop_ increased from 1 mg/ml to 5 mg/ml, the contents of N and C are increased, while the content of Ag decreased (Fig. S3c-Fig. S3f).

**Fig. 3:**
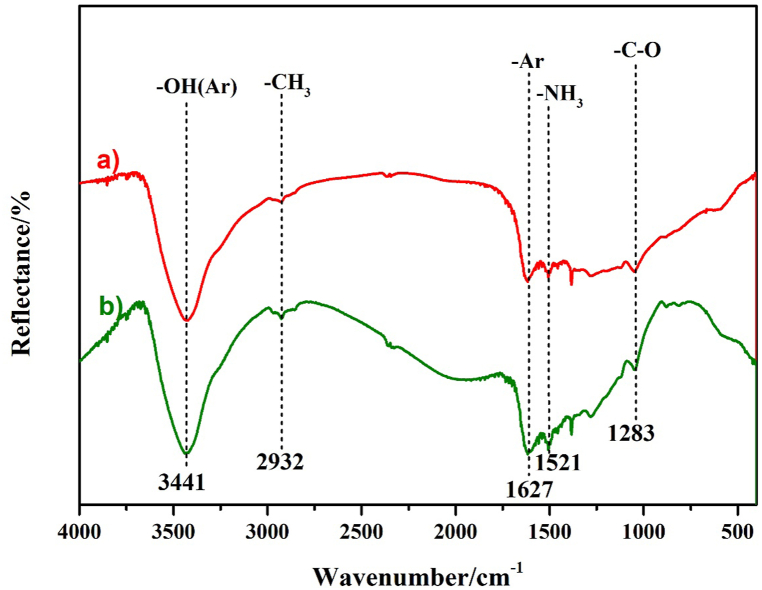
FT-IR spectra of a) Ag@PDA*1* film and b) Ag@PDA*5* film.

The Ag@PDA films were further characterized using FTIR. In our previous report, the FTIR spectrum of PDA film exhibited absorption peaks such as the phenolic hydroxyl stretching vibrations of the catechol group (3435 cm^−1^), the stretching vibrations of aromatic rings (1582 cm^−1^), and the N–H shearing vibration of the amide group (1521 cm^−1^) [36]. Fig. 3 shows FT-IR spectra of Ag@PDA*1* film and Ag@PDA*5* film. As depicted in Fig. 3a and b, and our previous report, the main absorption peaks of Ag@PDA*1* film, Ag@PDA*2* film, and Ag@PDA*5* film are almost consistent with those of PDA film, further indicating that PDA still exists in the Ag@PDA films. Additionally, the minor red shifts from 3435 cm^−1^ to 3441 cm^−1^ of the phenolic hydroxyl stretching vibrations and from 1582 cm^−1^ to 1627 cm^−1^ of the stretching vibrations of aromatic rings are probably attributed to the hydroxyl bond and inductive effects between Ag and phenolic hydroxyl groups.

**Fig. 4:**
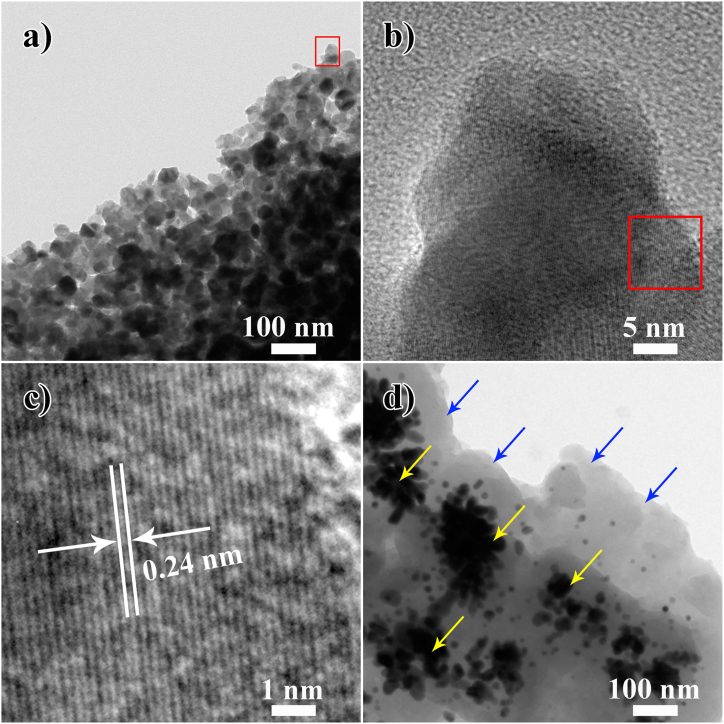
TEM images of a) Ag@PDA*1* film, b) enlarged image of the red rectangle in a), c) HRTEM image of the red rectangle region in b), and d) Ag@PDA*5* film. Ag NPs and PDA layers in the Ag@PDA5 film are pointed by the blue arrows in Fig. 4d, respectively.

Fig. 4 displays the TEM images of Ag@PDA*1* and Ag@PDA*5* films. As depicted in Fig. 4a, numerous inhomogeneous NPs with average diameter of 24 nm are presented in the Ag@PDA*1* film (Fig. S4a). The HRTEM image of the NPs (Fig. 4c, the enlarged image of the red rectangle region in Fig. 4b) reveals lattice fringes of 0.24 nm, which correspond to the (111) facet of metallic Ag, indicating that the NPs in the Ag@PDA*1* film are Ag NPs. In our previous report, it was demonstrated that Ag NPs and Ag aggregates in the Ag@PDA*2* film were coated with PDA layers [36]. However, in the Ag@PDA*1* film, a significant presence of Ag NPs is observed, with the shapeless PDA layers being scarcely visible, although the existence of PDA in the Ag@PDA*1* film has been confirmed by XPS and FTIR analysis. In comparison with the Ag@PDA*2* film, the Ag@PDA*5* film exhibits smaller, inhomogeneous Ag NPs with an average diameter of 12 nm (Fig. S4b), along with Ag aggregates (pointed by the yellow arrows in Fig. 4d) wrapped in thicker PDA layers (pointed by the blue arrows in Fig. 4d).

### 3.2 Formation mechanism of Ag@PDA film-modified NiTi alloy

The fabrication of PDA film on material in mild alkaline dopamine solution is a time-consuming process due to the low solubility of O_2_ in the solution. Meanwhile, dopamine possesses excellent reducing capacity [38,39]. When constructing the Ag@PDA film on NiTi alloy in the mixed solution of AgNO_3_ and dopamine, the Ag ions, with their strong oxidation capacity, could be instantaneously reduced to Ag NPs or Ag aggregates by a portion of dopamine. Subsequently, the residual dopamine underwent a time consuming oxidation and polymerization process on Ag NPs, Ag aggregates, and NiTi alloy to form an Ag@PDA film. Therefore, the Ag@PDA film-modified NiTi alloy can be constructed through the one-pot route. In this study, under fixed C_AgNO3_ and coating time, a lower C_dop_ could reduce the speed of nucleation and growth of Ag NPs. The lesser amount of residual dopamine in the mixed solution might not inhibit the growth of Ag NPs, leading to the formation of larger-sized Ag NPs and thinner PDA layers in the Ag@PDA*1* film. Increasing the C_dop_ could induce a higher number of Ag nuclei and result in the formation of smaller Ag NPs with higher surface energy. To minimize surface energy, the Ag NPs tend to aggregate [40,41] under moderate C_dop_ conditions. Therefore, the Ag@PDA*2* film contains a higher number of Ag NPs with smaller sizes aggregates compared to the Ag@PDA*1* film. Further increasing C_dop_ could reduce the aggregation tendency of Ag NPs and contribute to thicker PDA layers in the obtained Ag@PDA films. Thus, when C_dop_ is 5 mg/mL, independent Ag NPs and Ag aggregates are coated by thick PDA layers. This could explain the decrease in Ag content in the Ag@PDA films measured by XPS, attributed to the thickening PDA layers with increasing C_dop_. In contrast, when keeping C_dop_ and coating time constant, a lower C_AgNO3_ could consume less dopamine compared to a higher C_AgNO3_, resulting in an Ag@PDA film with thicker PDA layers. As C_AgNO3_ increasing, more dopamine in the mixed solution should be used to reduce Ag ions to Ag NPs, causing the PDA layers in the obtained Ag@PDA film to become progressively thinner [36].

**Fig. 5:**
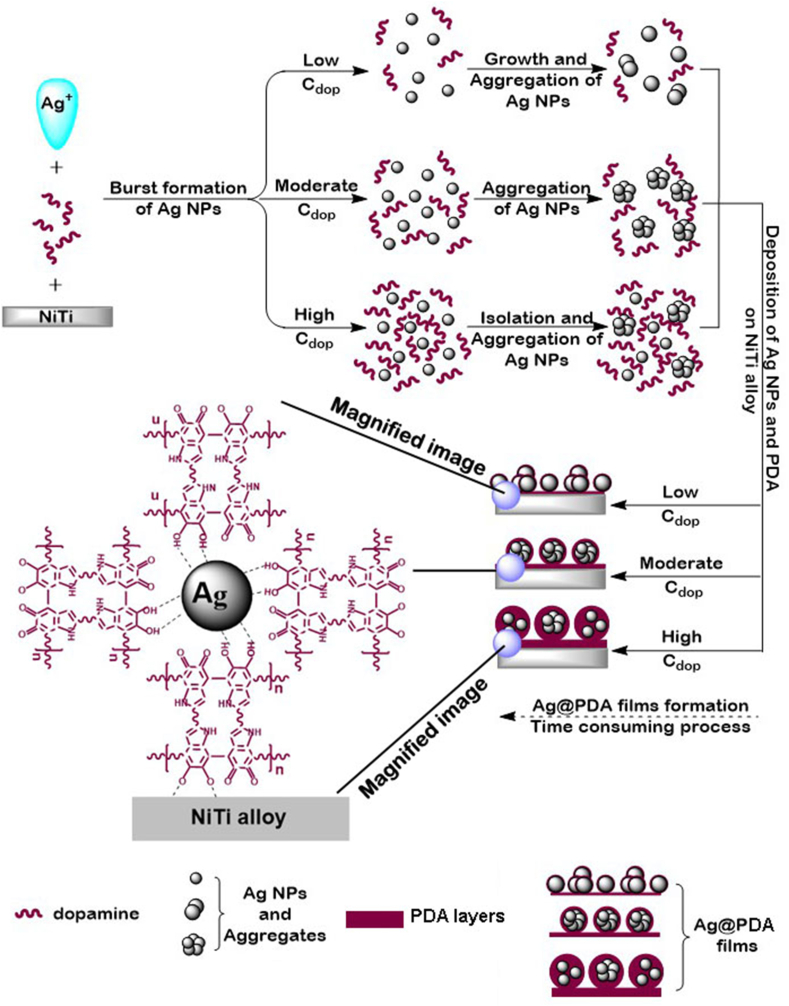
Schematic illustration of the formation mechanism of Ag@PDA films modified NiTi alloy by adjusting C_dop._

Based on the analysis above, we propose a potential mechanism for the one-pot construction of Ag@PDA film-modified NiTi alloy by adjusting C_dop_. Firstly, Ag NPs are instantaneously generated via a redox reaction between Ag ions and dopamine. Secondly, the Ag NPs undergo further grow or self-assembly at lower C_dop_ levels, self-assemble at moderate C_dop_ levels, and either isolate or self-assemble at higher C_dop_ levels. Thirdly, the residual dopamine polymerizes on the deposited Ag NPs or Ag aggregates, as well as NiTi alloy, resulting in the formation of Ag@PDA films. Fig. 5 illustrates the schematic representation of the formation mechanism of Ag@PDA film-modified NiTi alloy by adjusting C_dop_.

### 3.3 Corrosion resistance

**Fig. 6:**
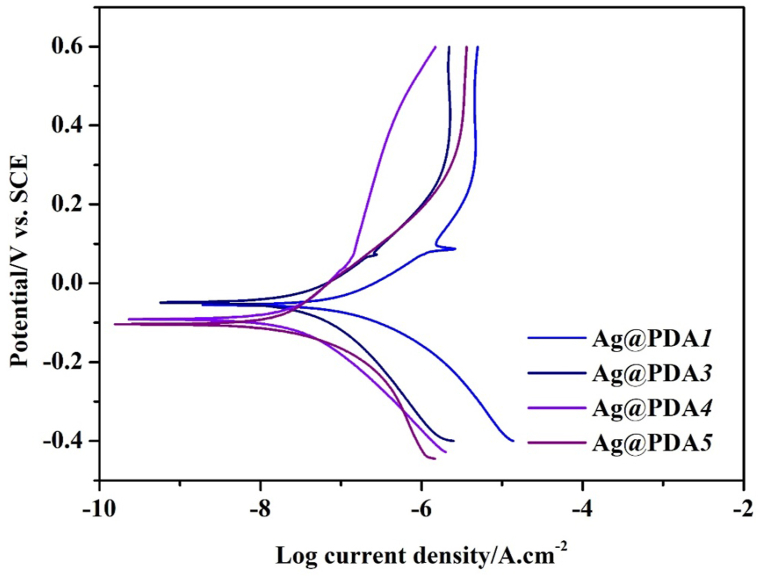
Polarization curves of Ag@PDA film-modified NiTi alloy in Hank's solution.

Fig. 6 depicts the polarization curves of Ag@PDA film-modified NiTi alloy in Hank's solution. In our previous study [36], the polished NiTi exhibited a low corrosion potential (E_corr_), a corrosion current density (I_corr_), and a pitting potential (E_pit_). In contrast, PDA film-modified NiTi alloy showed improved anticorrosion behavior evidenced by an increase in E_corr_ (−0.17 V) and a decrease in I_corr_ (1.15 × 10^−8^ A cm^−2^). Regarding Ag@PDA film-modified NiTi alloy, the Ag@PDA*2* displayed higher E_corr_ and I_corr_ than those of PDA film-modified NiTi alloy. As dipicted in Fig. 6, an increase in the C_dop_, resulted in a gradual decrease in both E_corr_ and I_corr_. Additionally, the small peaks in I_corr_ observed in the curves are attributed to the dissolution of Ag NPs during the polarization process. The corrosion results of polished NiTi alloy, PDA film-modified NiTi alloy, and Ag@PDA film-modified NiTi alloy are listed in Table S2.

In our experiment, prior to the deposition of PDA or Ag@PDA films, the NiTi alloy underwent mirror polished using diamond past. Luis et al. reported that the primary component on the NiTi surface is TiO_2_ after polishing with diamond paste, with a lower content of nickel oxide [42]. It has been documented that when the PDA film is deposited on the Ti surface, the abundant catechol groups in PDA film are capable of chelating with TiO_2_ [[43], [44], [45]]. Additionally, Kim et al. reported that the PDA film can firmly anchored on the surface of metallic Ni, although the specific binding style between Ni and PDA film was not specified [42]. Therefore, the PDA film can indeed be firmly anchored on the surface of the polished NiTi alloy. In our study, upon immersion of the PDA film-modified NiTi alloy in Hank's solution, the PDA film obstructs the contact between the NiTi alloy and the solution, thereby improving the corrosion resistance of the NiTi alloy. Conversely, when AgNO_3_ is mixed with dopamine solution, much catechol groups in the PDA film are oxidized by Ag ions, resulting in fewer catechol groups chelating with the surface of the NiTi alloy, thereby weakening the binding force between the Ag@PDA film and NiTi alloy. This weakened binding force could facilitate the intrusion of Hank's solution into the NiTi alloy, thereby increasing the I_corr_. Furthermore, in the mixed solution of AgNO_3_ and dopamine, more catechol groups than in pure dopamine solution are oxidized by Ag ions, leading to the formation of a PDA layer with more complete oxidation in the Ag@PDA film than in the pure PDA film. Therefore, the corrosion potential of Ag@PDA film-modified NiTi alloy is higher than that of PDA film-modified NiTi alloy. Additionally, the incorporation of Ag NPs with the strong electrode potential (E^θ^_Ag_ = 0.7996 V) [46] into the PDA layers might be another factor for improving the corrosion potential of Ag@PDA film-modified NiTi alloy.

### 3.4 Release behavior of Ni ions and Ag ions from Ag@PDA film-modified NiTi alloy

**Fig. 7:**
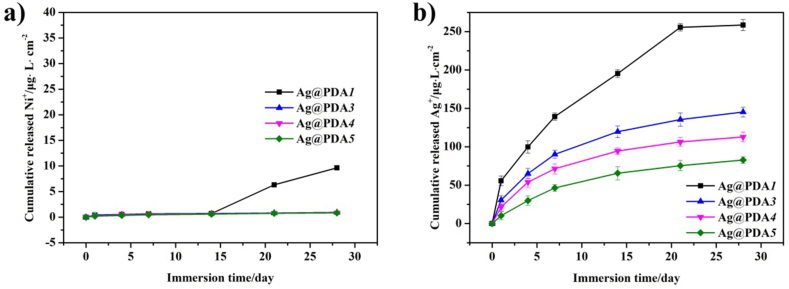
The release curves of a) Ni ions and b) Ag ions of Ag@PDA film-modified NiTi alloy in PBS solution with prolonging the immersion time.

Fig. 7a depicts the release curves of Ni ions from the Ag@PDA film-modified NiTi alloy with prolonged the immersion time in PBS solution. In our previous report, the polished NiTi alloy displayed a continual release of Ni ions and almost undetectable in PDA film-modified NiTi alloy and Ag@PDA*2* during the test [36]. As illustrated in Fig. 7a, the contents of Ni ions released from Ag@PDA*3*, Ag@PDA*4*, and Ag@PDA*5* are almost undetectable after immersion in PBS for 28 days. However, after 14 days of immersion, the Ni ions released from Ag@PDA*1* show a small increase compared to other Ag@PDA film-modified NiTi alloy, which might be ascribed to the poor coverage of Ag@PDA*1* films on the modified NiTi alloy.

The release behavior of Ag ions from Ag NPs-containing films plays an important role in influencing their antibacterial activity. Fig. 7b illustrates the time-dependent release of Ag ions from Ag@PDA film-modified NiTi alloy. In our previous report, Ag@PDA*2* exhibited a consistent release of Ag ions that persisted even after 28 days immersion [36]. By contrast, as depicted in Fig. 6b, the release of Ag ions from Ag@PDA*1* ceases after 21 days of testing, while other Ag@PDA film-modified NiTi alloy continue releasing ions beyond 28 days of immersion. Additionally, as depicted in Fig. 6b, during the initial 24 h, the Ag release rates of Ag@PDA film-modified NiTi alloy are decreased along with decrement of C_dop_. Hence, the release behavior of Ag ions can be controlled by adjusting the thickness of PDA layers in Ag@PDA films.

The PDA films or layers exhibit biodegradability, although the mechanism has not been fully illustrated. Jia et al. reported that the oligomers (dopamine2/5,6-dihydroxyindole) in PDA films degrade faster in soil than in water [47]. Chen et al. observed that PDA films dissolve in the form of fragments in alkaline solution [48]. Additionally, Sileika et al. [49], Saidin et al. [31] and Cheng et al. [50] have all reported that the coverage of PDA film on Ag NPs can reduce the dissolution rate of Ag ions and extend the release time. In this study, the Ag@PDA film was constructed via the one-pot route, where Ag NPs or Ag aggregates are wrapped in PDA layers. When immersed in PBS solution, the PDA layers in the Ag@PDA film come into contact with the solution first. As a portion of the PDA layers being dissolved, pores form, allowing the solution to contact the Ag NPs and subsequently dissolve Ag ions. With increasing C_dop_, a thicker PDA layer is formed in the Ag@PDA film, making the formation of pores more difficult and consequently lowering the dissolution rate of Ag ions.

### 3.5 Antibacterial performance

**Fig. 8:**
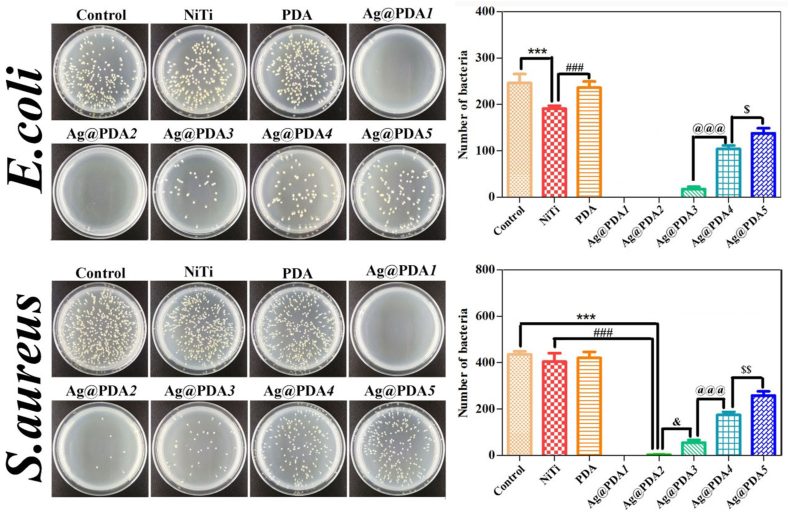
Typical colony forming and analytical results of *E. coli* and *S. aureus* incubated with the samples at 37 °C for 24 h. The data was obtained from the CFU counts (n = 3), Where *** represents P < 0.001 *vs.* Control, ### represents P < 0.001 *vs.* NiTi, & represents P < 0.05 *vs.* Ag@PDA2, @@@ represents P < 0.001 *vs.* Ag@PDA3, $$ represents P < 0.01 *vs.* Ag@PDA*4*, and $ represents P < 0.05 *vs.* Ag@PDA*4*, respectively.

The *E. coli* and *S. aureus* are common model pathogens to study the antibacterial properties of biomaterials [51,52]. Therefore, colony formation of the two strains was carried out to investigate the antibacterial performance of Ag@PDA film-modified NiTi alloy. Fig. 8 shows the typical colonies of *E. coli* and *S. aureus* along with the corresponding analytical results, which were obtained from the bacterial suspensions incubated with the samples at 37 °C for 24 h. The colony counts of *E.coli* and *S.aureus* in the NiTi and PDA (represent PDA film-modified NiTi alloy) groups depict a slightly reduction, with no statistically significant difference compared to the control group (P > 0.05), respectively, suggesting the poor antibacterial properties of NiTi and PDA film-modified NiTi alloy. In comparison, the colony count of Ag@PDA film-modified NiTi alloy significant decreased, and increased (P < 0.001) with the increment of C_dop_. The specific AR of the samples towards *E.coli* and *S.aureus* is listed in Table S3.

Ag NPs, possessing a broad spectrum of antibacterial properties, find extensive applications in clinical settings. The accepted antibacterial mechanism of Ag NPs entails the release of Ag ions from Ag NPs, which interact with bacterial cell walls and thiol groups, subsequently inactivating bacterial proteins and leading to bacteria death [53,54]. It has been demonstrated that the release of metal ions can be suppressed by the wrapped PDA layers [49]. In this study, Ag NPs or Ag aggregates in Ag@PDA films are wrapped by the PDA layers, effectively reducing the release rate of Ag ions. Demonstrating Ag release curves, it is observed that with an increase in the C_dop_, the release rate of Ag ions from the corresponding Ag@PDA film-modified NiTi alloy decreases, thereby weakening the antibacterial efficiencies within the initial 24 h. Conversely, with the thickening of the PDA layers, the time taken for the release of Ag ions from the Ag@PDA films on the modified NiTi alloy is delayed, thereby extending the antibacterial duration. Consequently, the Ag@PDA films, constructed by adjusting C_dop_, offered controllable antibacterial activities to the modified NiTi alloy.

### 3.6 *In vitro* and *In vivo* biosafety evaluation

**Fig. 9:**
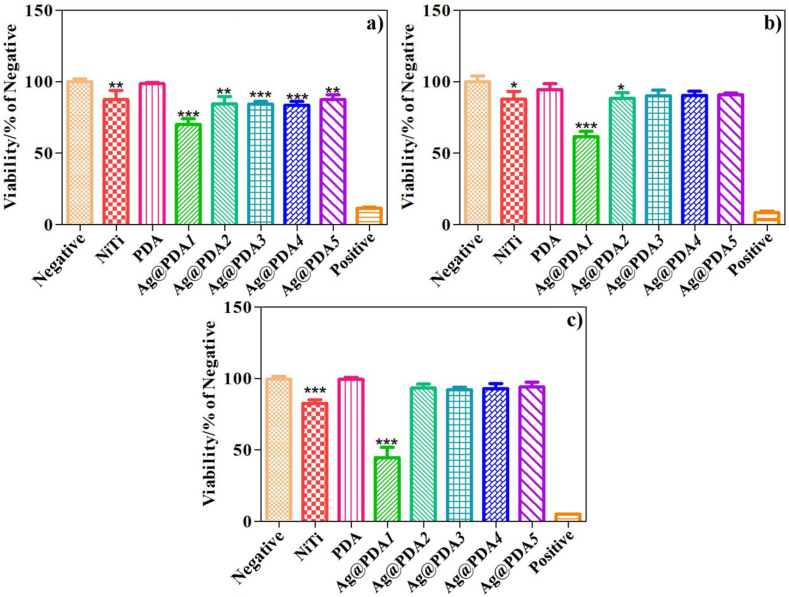
The *in vitro* viability of *hBMSCs* incubated with samples for a) 1 day, b) 3 days, and c) 5 days. Where * represents P < 0.05 vs. Negative, ** represents P < 0.01 vs. Negative, and *** represents P < 0.001 vs. Negative, respectively.

**Fig. 10:**
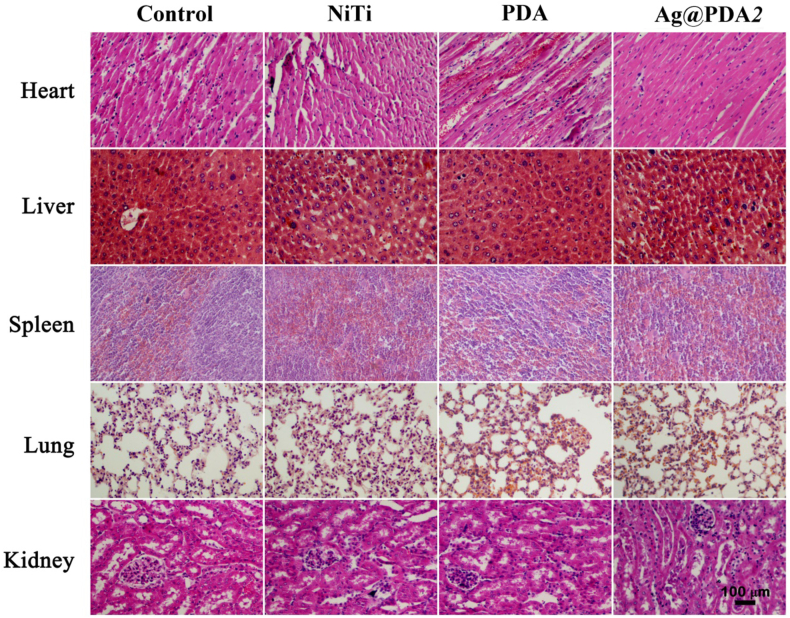
H&E staining of heart, liver spleen, lung, and kidney from control group, NiTi group, PDA group, and Ag@PDA*2* group.

The biosafety of biomaterials is a crucial requirement for their clinical applications. Therefore, the *in vitro* cytocompatibility of Ag@PDA film-modified NiTi alloy towards *hBMSCs* was evaluated using CCK8 assay. Fig. 9 depicts the viability of *hBMSCs* incubated with NiTi, PDA (represents PDA film-modified NiTi alloy), and Ag@PDA film-modified NiTi alloy for 1 day, 3 days, and 5 days. In comparision with the negative control group, the cell viability of NiTi and PDA exhibit slight decrease and remain stable during the testing period. However, Ag@PDA*1* exhibits a noticeable suppression of cell viability compared to other groups after 1 day of incubation (70 %, Fig. 9a). By the 5th day of incubation, the cell viability of Ag@PDA*1* further decreases to 45 % (Fig. 9c), indicating the cytotoxicity of Ag@PDA*1*. Comparatively, the cell viability of the other Ag@PDA film-modified NiTi alloy remains above 80 % after 1 day of incubation, about 85 % after 3 day of incubation (Fig. 9b), and reaches near 90 % after 5 days (Fig. 9c), indicating the desirable cytocompatibility. Furthermore, the long-term *in vivo* tissue biocompatibility of NiTi, PDA, and Ag@PDA*2* was evaluated. Major organs including the heart, liver, spleen, lung, and kidney were collected on day 30 for H&E staining. As illustrated in Fig. 10 and Fig. S5, no significant pathological changes or tissue damage were observed compared to the control group, further confirming that the implantation of Ag@PDA film-modified NiTi alloy exhibited satisfactory biosafety and did not induce any tissue toxicity.

Surplus Ag NPs have been demonstrated to be cytotoxic to several mammalian cell lines, including rat liver cells (BRL 3A) [55], male mouse germline cells (C18-4) [56], human lung fibroblast cells (IMR-90), and human glioblastoma cells [57]. The cytotoxicity of Ag NPs is primarily associated with the release of Ag ions and their exposure doses [58,59]. Despite the cytotoxicity levels of both Ag NPs and Ag ions being much higher than those required for antibacterial purposes [58], special precautions should be taken before utilizing Ag NPs as an antibacterial agent. Studies have shown that a PDA film-modified surface can promote the normal growth of mammalian cells without inducing cytotoxicity effects [[60], [61], [62]]. In Ag@PDA films, the Ag NPs or Ag aggregates are wrapped in PDA layers. Thicker PDA layers, such as those in Ag@PDA*2* or Ag@PDA*5*, can directly contact cells, thereby reducing the exposure Ag NPs, inhibiting the release of Ag ions, and consequently reducing cytotoxicity. However, PDA layers are barely observed in Ag@PDA*1*, indicating that thinner PDA layers do not fully coat the Ag NPs or Ag aggregates. This incomplete coating could resulte in the rapid release of Ag ions and notable cell cytotoxicity in Ag@PDA*1*. Additionally, it has been demonstrated that Ag toxicity occurs at serum levels as low as 300 μg L^−1^, manifesting as argyria, leukopenia, and alterations in renal, hepatic, and neural tissues [50]. In our study, the maximum cumulative concentrations of Ag ions released from Ag@PDA*1* were approximately 250 μg L^−1^, which is below the serum toxicity threshold induced by Ag ions. Furthermore, biological fluids could dilute the subsequent release of Ag ions when the samples are used in clinical applications, potentially attenuating the cytotoxic effects induced by Ag ions. Therefore, during the *in vivo* implantation test period, no obvious pathological changes or tissue damage were observed in the main organs.

## 4 Conclusion

In this study, Ag@PDA film-modified NiTi alloy were constructed via a one-pot route in a mixed solution of AgNO_3_ and dopamine. By fixing the concentration of AgNO_3_ and coating time, and varying the concentration of dopamine, polydopamine layers with adjustable thickness was incorporated into the Ag@PDA films. This conferred the modified NiTi alloy with enhanced corrosion resistance, controllable release of Ag ions, and thereby, controllable antibacterial activities. Specifically, Ag@PDA film-modified NiTi alloy with thicker polydopamine layers exhibited weaker short-term antibacterial efficacy (24 h) but demonstrated prolonged antibacterial duration. Conversely, thinner polydopamine layers in Ag@PDA films provided the modified NiTi alloy with stronger short-term antibacterial efficacy (24 h) but shorter antibacterial duration. Additionally, the presence of polydopamine layers on the Ag NPs or Ag aggregates contributed to satisfactory biosafety of the Ag@PDA film-modified NiTi alloy as implants. Therefore, the construction of Ag@PDA films on NiTi alloy could represent and ideal approach to inhibit the leaching of Ni ions and address various types of infections associated with NiTi alloy implants.

## Data availability statement

The data are available from the corresponding author on reasonable request.

## Ethics declarations

All animal experiments adhered to the guidelines set forth by the Ethics Committee on Animals of Mudanjiang Medical University (Mudanjiang, China) (approval number: IACUC-20220516-9).

## CRediT authorship contribution statement

**Ying Li:** Writing – original draft, Methodology, Investigation, Formal analysis, Data curation. **Yongkui Yin:** Writing – review & editing, Supervision, Project administration, Funding acquisition, Conceptualization. **Luxin Li:** Validation, Supervision, Investigation.

## Declaration of competing interest

We declare that we do not have any competing financial interests, associative interests, or personal relationships that could have appeared to influence the work in this paper.
