# Supplementary material for: Conferring NiTi alloy with controllable antibacterial activity and enhanced corrosion resistance by exploiting Ag@PDA films as a platform through a one-pot construction route
Source: Heliyon. 2024 Jul 9;10(14):e34154. doi: 10.1016/j.heliyon.2024.e34154 (PMC11304019; doi:10.1016/j.heliyon.2024.e34154)
Supplement: Multimedia component 1 [file mmc1.docx]

**Table S1** Detailed surface element contents of polished NiTi alloy, PDA film-modified NiTi alloy, and Ag@PDA film-modified NiTi alloy constructed by adjusting C_dop_

| Sample | C (at.%) | O (at.%) | N (at.%) | Ag (at.%) | Ni (at.%) | Ti (at.%) |
| --- | --- | --- | --- | --- | --- | --- |
| NiTi | 44.36 | 38.74 | - | - | 4.03 | 12.87 |
| PDA | 78.89 | 14.38 | 6.73 | - | - |  |
| Ag@PDA*1* | 76.01 | 13.72 | 7.88 | 0.89 | 0.14 | 1.34 |
| Ag@PDA*2* | 75.28 | 17.53 | 6.57 | 0.62 | - | - |
| Ag@PDA*3* | 75.09 | 17.71 | 6.9 | 0.3 | - | - |
| Ag@PDA*4* | 75.37 | 18.29 | 6.35 | < 0.1 | - | - |
| Ag@PDA*5* | 75.69 | 17.8 | 6.5 | < 0.1 | - | - |

**Table S2** Corrosion results of polished NiTi alloy, PDA film-modified NiTi alloy, and Ag@PDA film-modified NiTi alloy in Hank’s solution

| Samples | E_corr_/V vs. SCE | I_corr_/A·cm^-2^ | E_pit_/V |
| --- | --- | --- | --- |
| NiTi | -0.33 | 1.45E-7 | 0.38 |
| PDA | -0.17 | 1.15E-8 |  |
| Ag@PDA*1* | -0.05 | 1.07E-7 |  |
| Ag@PDA*2* | -0.08 | 5.84E-8 |  |
| Ag@PDA*3* | -0.05 | 3.71E-8 |  |
| Ag@PDA*4* | -0.09 | 2.63E-8 |  |
| Ag@PDA*5* | -0.09 | 1.86E-8 |  |

**Table S3** The specific AR of the samples towards *E.coli* and *S.aureus*.

|  | *E.coli* (AR) | *S. aureus* (AR) |
| --- | --- | --- |
| NiTi | 2.96% | 0.07% |
| PDA | 0.04% | 0.06% |
| Ag@PDA*1* | 100% | 100% |
| Ag@PDA*2* | 100% | 99.32% |
| Ag@PDA*3* | 92.34% | 87.21% |
| Ag@PDA*4* | 56.85% | 58.68% |
| Ag@PDA*5* | 45.56% | 41.55% |


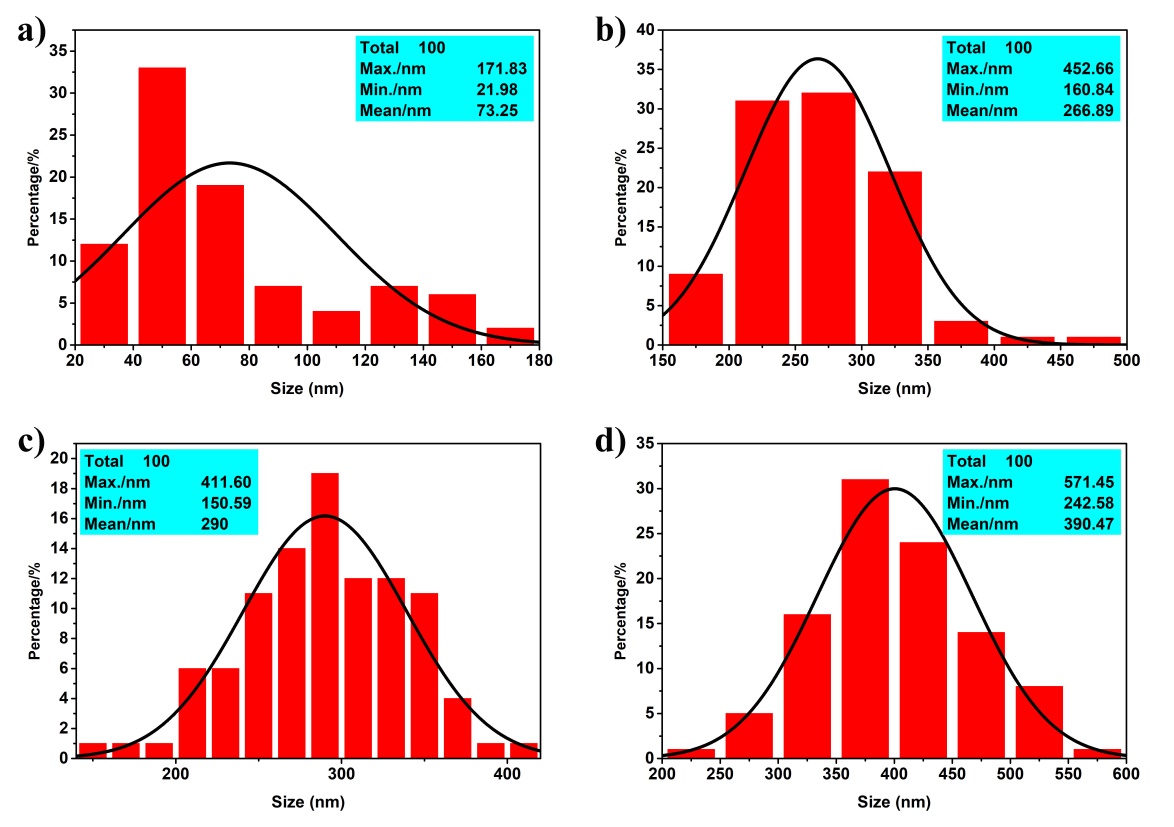


**Fig.S1** Size distributions of Ag NPs or Ag aggregates in a) Ag@PDA*1*, b) Ag@PDA*3*, c) Ag@PDA*4*, and Ag@PDA*5*, respectively.


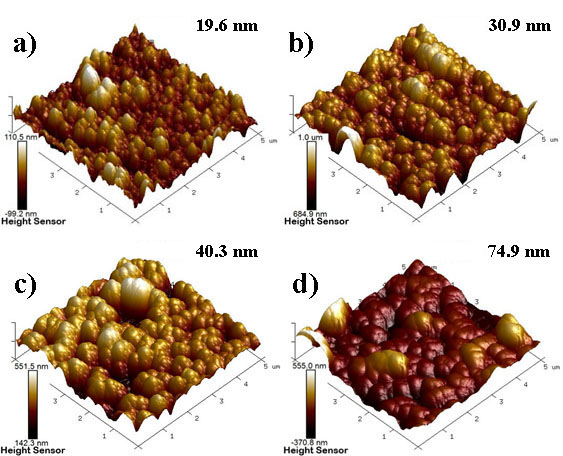


**Fig.S2** The AFM images of Ag@PDA film modified NiTi alloy, a) Ag@PDA*1*, b) Ag@PDA*3*, c) Ag@PDA*4*, and d) Ag@PDA*5.*


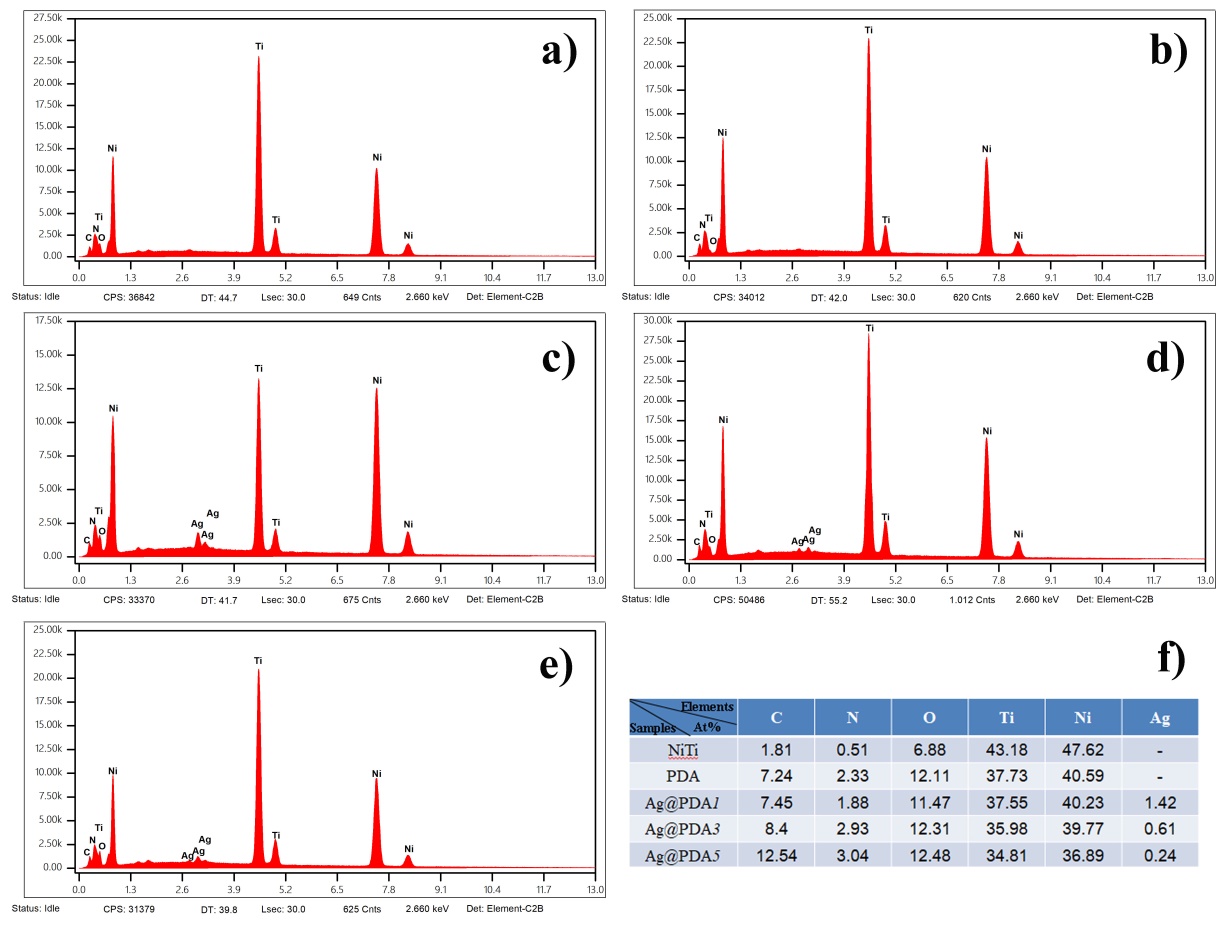


**Fig.S3** EDAX spectra and chemical constituents of the samples, a) NiTi, b) PDA, c) Ag@PDA*1*, d) Ag@PDA*3*, e) Ag@PDA*5*, and f) chemical constituents obtained by EDAX.


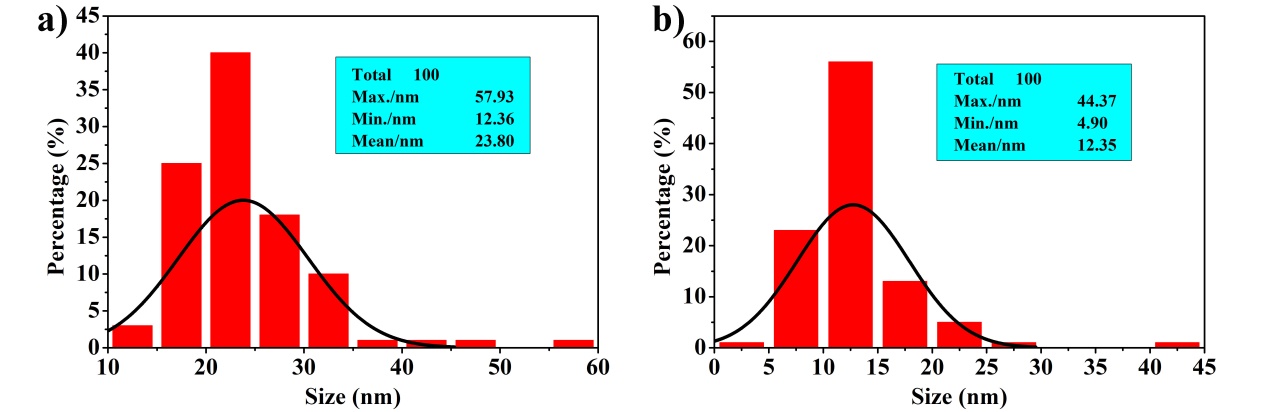


**Fig. S4** Size-distribution of Ag NPs in a) Ag@PDA*1* film and b) Ag@PDA5 film, respectively.


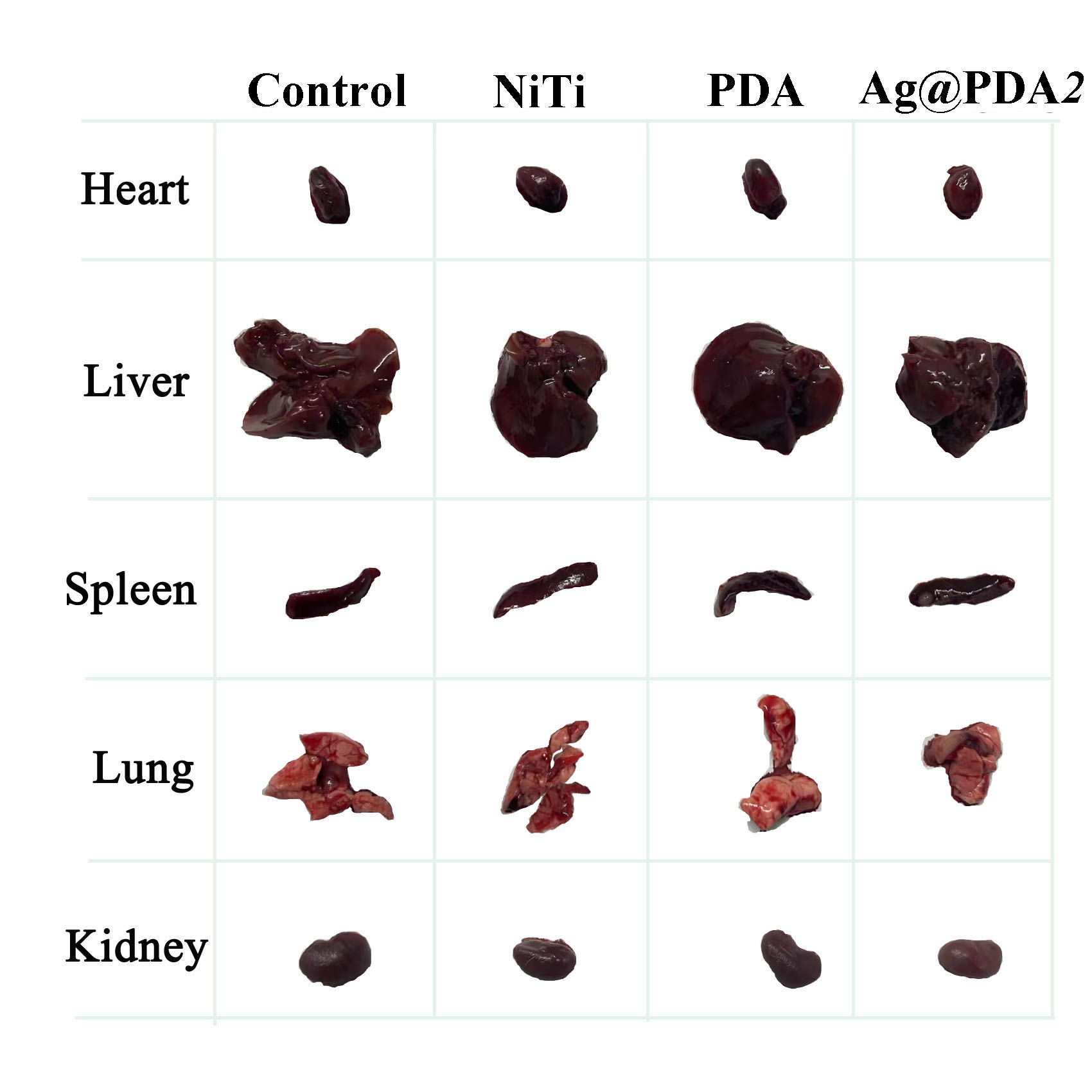


**Fig.S5** Representative photographs of heart, liver spleen, lung, and kidney from different groups.
